# Proceedings of the Annual Meeting of the Æsculapian Society

**Published:** 1856-12

**Authors:** 


### MEDICAL INTELLIGENCE.

*Proceedings of the Annual Meeting of the Æsculapian Society, held at Grand View, Ill. Oct. 29th and* 30*th*, 1856.

The Æsculapian Society met in annual session at Grand View, Oct. 29th. In the absence of the President (Dr. York,) the Vice-President, Dr. Stormont, took the Chair, and called the Society to order at 11 o'clock.

The minutes of the last meeting were read and approved.

On motion, the regular order of business was suspended. Dr. Chambers then announced the death of Dr. E. C. Banks, one of the founders of the Society; and, after paying a feeling tribute to the professional and personal worth of the deceased, offered the following resolutions, which were unanimously adopted, viz.:—

*Resolved*, That in the death of Dr. Elijah Columbus Banks, this Society acknowledges a Providential visitation, which has removed from their fellowship an esteemed associate and co-worker in all that tends to elevate the profession to which they belong.

*Resolved*, That, as members of the medical profession, we express our sense of the loss we have sustained in the death of one of its members, who, by his natural gifts, his acquirements, his zeal, his industry and his devotion to his calling, has raised the standard of our noble profession, and commanded the confidence of his professional brethren throughout the surrounding country.

*Resolved*, That the heartfelt sympathies of this Society be respectfully tendered to the family of the deceased, and that while we assure them their sorrow is shared by all around them, we trust they may find consolation in the many good works which he did while with us; in the gratitude with which his name will be cherished, and in the trust that a useful earthly existence has prepared him for a life of clearer knowledge and purer happiness.

*Resolved*, That a copy of these resolutions be sent to Mrs. Banks, by the Secretary.

On motion of Dr. Davis, the Society then adjourned to meet at one o'clock.

AFTERNOON SESSION.

The President, Dr. York, called the Society to order according to adjournment.

Drs. Davis and McCord were appointed to fill vacancies in the Board of Censors.

Dr. Davis proposed Dr. Frizell, of Bloomfield, for membership. Referred to the Censors.

Reports from standing committees called for.

Dr. Chambers, from the Committee on Surgery, read a report. Discussed by Drs. Davis, Chambers, York, and others.

Dr. Payne, Chairman of the Board of Censors, reported favorably on the application of Dr. Frizell. The Doctor then read a paper on Inflammation of the Uterus, which was discussed by Drs. F. R. Payne, Davis, York, H. R. Payne, Chambers, McCord, Frizell, and Stormont.

Dr. Frizell was then balloted for and unanimously elected.

Dr. Davis read a paper on Pneumonia. Discussed by Drs. Chambers, Davis, Mc Cord, and York.

Dr. McCord asked to be excused for not reporting on Typhoid Fever. Excused. Some pertinent remarks on the diagnosis of this disease were made by Drs. York, Chambers, and Payne.

The Society then proceeded to the election of officers for the ensuing year, which resulted as follows, viz.:—

Dr. F. R. PAYNE, *President*.

Dr. W. M. CHAMBERS, *Vice-President*.

Dr. D. W. STORMONT, *Secretary*.

Dr. D. O. MCCORD, *Treasurer*.

Drs. S. York, T. M. Smith, J. M. Stuh, S. W. Thompson, and H. R. Payne, *Censors.*

On motion, the Society then went into an election for Delegates to the American Medical Association and to the State Society. Drs. S. York and D. W. Stormont were elected Delegates to the National Association, with power to appoint alternates; and Drs. Duncan, Stormont, Chambers, and Smith were elected Delegates to the State Society, with power to appoint alternates.

On motion of Dr. Stormont, the Society now adjourned to meet at the Presbyterian Church, at 6½ o'clock, to hear the Annual Address by Dr. H. R. Payne.

EVENING SESSION.

The Society met as per adjournment. Dr. H. R. Payne, being introduced, delivered an excellent address to a large and attentive audience on the True Position of the Medical Profession. Dr. York, the retiring President, then delivered a spirited Valedictory on Quackery in the Profession. The audience was then dismissed, and the Society proceeded to the regular business.

On motion of Dr. York, the Treasurer was instructed to make a full report of the condition of the finances of the Society, at the next meeting.

Dr. Stormont moved that the thanks of the Society be tendered to Drs. Payne and York for their very excellent addresses, and copies requested for publication. Carried.

The resolution offered by Dr. Mitchell, at the last meeting, coming up, on motion of Dr. York it was laid on the table.

Charleston was selected as the place for holding the next meeting.

Dr. H. W. Davis was appointed to deliver the next public address.

The following gentlemen were appointed Chairmen of the Standing Committees (of their own selection,) to report on the subjects annexed to their names, at the next *annual* meeting, viz.:—

Dr. S. Thompson, Surgery.

Dr. F. R. Payne, Practical Medicine.
" J. Tenbrook, Midwifery.
" S. W. Thompson, Epidemics.
" C. A. Hunt, Indigenous Botany.

Drs. McCord, Smith, and Frizell were appointed a Business Committee, to select subjects for essays and reports at the next semi-annual meeting, said Committee to report to-morrow morning.

On motion the Society adjourned to meet to-morrow morning, 8 o'clock.

OCT. 30, MORNING SESSION.

The Society met as per adjournment, Dr. Payne in the chair.

The Secretary read a report on Epidemics, by Dr. Chambers.

Dr. H. R. Payne read a paper on Malignant Inflammation of the Fauces, reporting several cases.

The Business Committee announced the following subjects, and the President appointed the gentlemen whose names are opposite the subjects, to report thereon at the next meeting, viz.:—

Epidemic Opthalmia, Dr. S. York.
Milk Sickness, Dr. J. M. Logan.
Scrofula, Dr. T. D. Washburn.
Pneumonia, Dr. H. W. Davis.
Rheumatism, Dr. T. M. Smith.
Prolapsus Uteri, Dr. Balch.
Veratrum Viride, Dr. C. Gorham.
Epidemic Dysentery, Dr. D. Bowers.
Acute Bronchitis, Dr. J. D. Mitchell.
Use and Abuse of Alcohol, Physiologically Considered, Dr. J. M. Hinkle.
Stomatitis Materna, Dr. W. M. Chambers.
Delirium Tremens, Dr. Holms.
Relations between Cardiac and Pulmonary Diseases, Dr. J. H. Frizell.
Sequalæ of Intermittent Fever, Dr. D. O. McCord.
Influence of Malaria in Modifying Diseases, Dr. H. R. Payne.
Membranous Croup, Dr. C. Johnson.

Typhoid Fever, its Diagnosis and Treatment, Dr. C. M. Hamilton.

Cause of Intermittent Fever, Dr. V. R. Bridges.

Induration, Inflammation, and Ulceration of the Os Uteri, and Use of Speculum, Dr. Davis.

Best Means of Procuring Water for Drinking Purposes, Dr. O. Q. Herrick.

On motion of Dr. Chambers, Drs. S. Thompson, Bridges, and Bowers were continued a Committee on Substitutes for Quinine, to report at the next meeting.

Dr. A. A. Lodge, of Midway, being proposed and recommended by the Censors, was unanimously elected a member of the Society.

Dr. Chambers moved that a committee be appointed to memorialize the Legislature to pass a law regulating the sale of Patent Medicines. Carried. Drs. Chambers, Davis, and York were appointed said committee.

The Society then adjourned to meet at one o'clock.

AFTERNOON SESSION.

The Society met, the President in the Chair.

Dr. Payne moved that the Treasurer be instructed to discharge all the just debts of the Society. Carried.

Dr. Chambers moved the Treasurer call on each member for two dollars assessment. Carried.

Here quite a lengthy discussion arose on the propriety of publishing, semi-amnually, all the transactions of the Society; but the subject was laid over without any definite action being taken on it.

Dr. Chambers offered the following resolution, which was adopted, viz.:—

*Resolved*, That the Secretary be instructed to correspond with the Secretary of the Smithsonian Institute, in reference to procuring four full sets of Meteorological Instruments for the use of the Society.

Drs. Chambers, Lodge, and Herrick, were appointed a Committee of Arrangements.

It was ordered that a copy of the proceedings of this meeting be furnished the *North-Western Medical and Surgical Journal*, and the newspapers of the district, for publication.

The Society then adjourned to meet in Charleston, on the last Wednesday in May.

F. R. PAYNE, *President*.

D. W. STORMONT, *Secretary*.

